# The Non-High-Density Lipoprotein Cholesterol (Non-HDL-C) to HDL-C Ratio (NHHR) and Its Association with Chronic Kidney Disease in Chinese Adults with Type 2 Diabetes: A Preliminary Study

**DOI:** 10.3390/nu17071125

**Published:** 2025-03-24

**Authors:** Xiangyu Chen, Mingbin Liang, Jie Zhang, Chunxiao Xu, Lijin Chen, Ruying Hu, Jieming Zhong

**Affiliations:** Department of Non-Communicable Disease Control and Prevention, Zhejiang Provincial Center for Disease Control and Prevention, Hangzhou 310051, China; xychen@cdc.zj.cn (X.C.); mbliang@cdc.zj.cn (M.L.); jiezhang@cdc.zj.cn (J.Z.); chxxu@cdc.zj.cn (C.X.); ljch@cdc.zj.cn (L.C.); ryhu@cdc.zj.cn (R.H.)

**Keywords:** type 2 diabetes mellitus, high-density lipoprotein cholesterol, non-high-density lipoprotein cholesterol, chronic kidney disease

## Abstract

**Objectives**: The objective of this study was to examine the association between non-high-density lipoprotein cholesterol (non-HDL-C) to high-density lipoprotein cholesterol (HDL-C) ratio (NHHR) and chronic kidney disease (CKD) in Chinese adults with type 2 diabetes mellitus (T2DM). **Methods**: This study originated from a survey carried out in Zhejiang Province, located in eastern China, between March and November 2018. To explore the relationship between NHHR and CKD, a multivariable logistic regression model was employed. The dose–response relationship was assessed using restricted cubic spline (RCS) analysis, while generalized additive models (GAMs) were applied to examine the associations between NHHR and urinary albumin-to-creatinine ratio (UACR) as well as estimated glomerular filtration rate (eGFR). Subgroup analyses were performed across various demographic and clinical categories to assess the consistency of the NHHR–CKD association. The optimal NHHR cutoff for CKD diagnosis, its predictive accuracy, and its comparison with its components and HbA1c were determined through receiver operating characteristic (ROC) curve analysis. **Results**: The study enrolled 1756 participants, including 485 individuals with CKD and 1271 without CKD. Multivariable logistic regression revealed a significant positive association between NHHR and CKD, with each standard deviation (SD) increase in NHHR linked to a 23% higher odds of CKD (OR = 1.23, 95% CI: 1.09–1.37) after adjusting for potential confounders. When comparing quartiles, the fully adjusted ORs for Q2, Q3, and Q4 were 1.29 (0.92–1.79), 1.31 (0.94–1.83), and 1.87 (1.34–2.60), respectively, relative to Q1 (*p* for trend < 0.01). RCS analysis confirmed a linear dose–response relationship between NHHR and CKD in both sexes (*p* for nonlinearity > 0.05). GAMs indicated a significant positive correlation between NHHR and UACR (ρ = 0.109, *p* < 0.001) but no significant association with eGFR (ρ = −0.016, *p* = 0.502). Subgroup analyses demonstrated consistent associations across most subgroups, except for the 18–44 years age group, the well-controlled glycemic group, and the non-alcohol drinking group (*p* > 0.05). ROC curve analysis identified an optimal NHHR cutoff of 3.48 for CKD prediction, with an area under the curve (AUC) of 0.606 (95% CI: 0.577–0.635). Notably, NHHR outperformed its individual components and HbA1c in predictive performance. **Conclusions**: This study revealed a linear link between higher NHHR levels and increased CKD prevalence in Chinese T2DM patients. NHHR may also serve as a potential complementary biomarker for early CKD detection, though further prospective studies are needed to confirm its predictive value and clinical utility in high-risk T2DM populations.

## 1. Introduction

The prevalence of type 2 diabetes mellitus (T2DM) has risen sharply globally, with China facing a particularly severe burden [1]. Recent epidemiological data reveal that 12.4% of the adult population in mainland China are living with diabetes, and the majority of these cases are classified as T2DM [2]. In addition to being a metabolic condition, T2DM, a metabolic disorder, significantly increases the risk of systemic complications, particularly cardiovascular disease and chronic kidney disease (CKD) [3]. These associated conditions are major drivers of elevated morbidity and mortality rates among individuals with T2DM [4,5].

CKD is one of the most serious complications associated with T2DM, marked by progressive albuminuria, a reduction in glomerular filtration rate, and an increased risk of cardiovascular events [6]. In China, over the past three decades, the absolute count of deaths and disability-adjusted life years associated with CKD linked to diabetes has shown a consistent upward trend [7]. Considering the chronic and typically irreversible trajectory of CKD, implementing timely detection methods for high-risk populations and adopting focused intervention approaches are critical to decelerating the advancement of the condition and enhancing long-term health results for affected individuals.

In patients with T2DM, lipid abnormalities play a significant role in the development and progression of CKD [8]. Among the lipid markers, high-density lipoprotein cholesterol (HDL-C) has protective effects on vascular and renal health due to its anti-inflammatory, antioxidant, and cholesterol reverse transport functions [9]. Low HDL-C levels are associated with an increased risk of CKD, potentially due to reduced endothelial protection and impaired renal function [10,11]. However, the relationship between HDL-C and clinical outcomes is complex and likely nonlinear, as evidenced by studies showing diminishing protective effects at higher HDL-C levels [12]. Moreover, interventions to raise HDL-C have failed to demonstrate clinical benefits [13]. Conversely, non-high-density lipoprotein cholesterol (non-HDL-C), calculated as total cholesterol (TC) minus HDL-C, represents the total atherogenic lipoprotein burden, including low-density lipoprotein cholesterol (LDL-C), very-low-density lipoprotein cholesterol (VLDL-C), and lipoprotein(a) [14]. Elevated non-HDL-C levels have been linked to a decline in estimated glomerular filtration rate (eGFR) and an increased risk of proteinuria, suggesting that excessive atherogenic lipids contribute to endothelial dysfunction, inflammation, and glomerular injury, thereby accelerating CKD progression in T2DM patients [15].

The non-HDL-C/HDL-C ratio (NHHR) reflecting the balance between protective and atherogenic lipoproteins, has been linked to endothelial dysfunction and inflammation, key pathways in CKD progression [16]. Currently, some evidence suggests that specific lipid ratios may better reflect lipid-related renal risk compared to traditional lipid parameters [17,18]. Similarly, NHHR has also emerged as a potential predictor of atherosclerotic cardiovascular disease and metabolic disorders [19]. However, the validity of such ratios is debated, particularly given the nonlinear effects of HDL-C and the lack of evidence supporting their superiority in guiding individual treatment decisions [20,21].

Given that T2DM involves chronic inflammation, insulin resistance (IR), and vascular dysfunction [22], lipid ratios like NHHR may offer insights into systemic metabolic risks that contribute to CKD alongside traditional renal markers like urinary albumin-to-creatinine ratio (UACR) and eGFR. While UACR and eGFR are cost-effective and directly reflect renal damage, lipid ratios could potentially identify at-risk individuals earlier by capturing dyslipidemia-related pathways, though their added value remains unproven. Our study explores NHHR’s association with CKD to determine if it complements the existing clinical tools, especially among high-risk groups, such as Chinese T2DM patients.

To address this gap in understanding, our study employs a cross-sectional dataset to examine the relationship between NHHR and CKD in Chinese adults diagnosed with T2DM. By elucidating this association, our results have the potential of contributing to a deeper understanding of the metabolic-renal interface and informing risk stratification strategies in clinical practice.

## 2. Materials and Methods

### 2.1. Study Subjects

The research subjects were drawn from a comprehensive investigation focused on complications associated with T2DM, conducted between March and November 2018 in Zhejiang Province, eastern China. The study included local residents aged 18 years or older who had been diagnosed with T2DM and recorded in the local health information platform.

A multi-stage randomized sampling strategy was employed to select participants. In stage one, two districts and two counties were randomly chosen from the province. During the second stage, four townships or subdistricts were randomly selected from each of the previously chosen districts and counties. In stage three, a random sample of 120 individuals diagnosed with T2DM was selected from each township or subdistrict, ensuring a balanced distribution across sex and age categories. This approach led to the inclusion of 1920 participants in the study. Further details regarding the study design and methodology are available in the published protocol [23].

**Figure 1:**
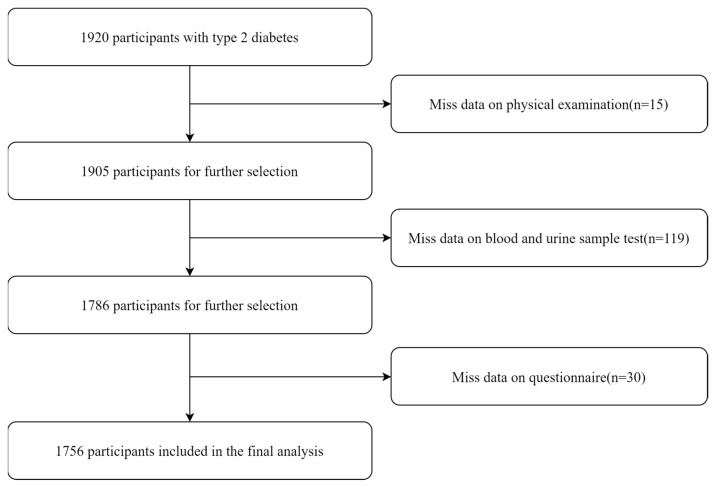
The detailed procedure of participant selection.

Following the application of exclusion criteria, 164 individuals were excluded, leaving a final analytical sample of 1756 participants (Figure 1).

### 2.2. Sample Size Calculation

The determination of the required number of participants was derived using the equation N = μ^2^ × *p* × (1 − *p*)/d^2^. Within this equation, the variables were specified as follows: *p* denoted the estimated proportion of CKD cases among Chinese individuals with T2DM, set at 0.271 [24]; μ was assigned a value of 1.96; and the relative error (d) was fixed at 0.05. Utilizing these parameters, the necessary sample size for each subgroup was calculated to be 304 individuals. Considering the division of the target population into 4 subgroups (urban and rural regions, along with sex) and anticipating a non-participation rate of 15.0%, the total number of subjects needed for the research was projected to be 1430. Consequently, the participant count in this study was deemed sufficient.

### 2.3. Data Collection

In this investigation, participants were required to complete a face-to-face questionnaire, undergo physical examinations (e.g., height, weight, and blood pressure), and provide fasting blood and urine samples for laboratory analyses. These analyses encompassed assessments of hemoglobin A1c (HbA1c), fasting plasma glucose (FPG), lipid profiles, renal function markers (urea, creatinine, and serum uric acid [SUA]), urinary creatinine and urinary albumin, etc. All procedures were conducted by trained personnel from township health centers, who had substantial professional experience and had undergone specific training for this research initiative.

Standardized instruments were utilized for anthropometric measurements: height was measured using a stadiometer, weight with an electronic scale (HD-390, TANITA, Tokyo, Japan), and waist circumference with a flexible retractable tape. Blood pressure and heart rate were recorded using an automated blood pressure monitor (HBP-1300, OMRON, Kyoto, Japan).

Fasting blood samples were collected from all participants after a 10–12 h overnight fast, along with first-morning urine specimens. FPG levels were assessed at local laboratories that had met qualification standards, employing either the glucose oxidase or hexokinase method. The remaining blood and urine samples were processed on-site, where they were centrifuged, aliquoted, and stored according to standardized preservation protocols. These samples were then transported by a logistics provider designated by the national project team for centralized analysis. HbA1c levels were measured using high-performance liquid chromatography (D10, Bio-Rad, Berkeley, CA, USA). Lipid profiles, including triglycerides (TG), TC, HDL-C, and LDL-C, as well as SUA and creatinine levels in blood and urine, were determined enzymatically (Cobas C701, Roche, Basel, Switzerland). Urinary albumin concentrations were quantified using an immunoturbidimetric method (Cobas C701, Roche, Basel, Switzerland).

### 2.4. Definitions of Variables

#### 2.4.1. Definitions of CKD and NHHR

CKD was diagnosed based on two primary criteria: the presence of albuminuria, defined as a UACR exceeding 30 mg/g; or impaired kidney function, characterized by an eGFR below 60 mL/min per 1.73 m^2^ [25,26]. The eGFR values were calculated utilizing the chronic kidney disease epidemiology collaboration equation [27]. Additionally, the NHHR was calculated by dividing the non-HDL-C concentration by the HDL-C concentration [28]. Non-HDL-C levels were obtained by subtracting HDL-C values from TC measurements.

#### 2.4.2. Definitions of Covariates

Hypertension was characterized by systolic blood pressure readings of 140 mmHg or higher, diastolic blood pressure of 90 mmHg or above, combined with a self-reported diagnosis of hypertension documented by medical institutions [29]. The body mass index (BMI) was calculated as weight (kg) divided by height squared (m^2^) and was categorized as <24 kg/m^2^ and ≥24 kg/m^2^ for the subgroup analysis [30]. Elevated glycemic markers were defined as HbA1c levels of 7.0% or more or FPG concentrations of 7.0 mmol/L or higher [31]. Educational background was categorized into three groups: secondary education or less, senior high school completion, and attainment of college education or higher. Marital status was divided into two classifications: married and other (including single, divorced, or widowed). Participants were stratified by age into young adults (18–44 years), middle-aged adults (45–59 years), and older adults (60 years and above). Residential status was classified as urban or rural based on the geographic location of participants’ homes. Routine exercise was defined as engaging in exercise for a minimum of 30 min per day, at least five days per week [32]. Smoking status was determined by current daily or occasional cigarette use, while alcohol drinking was recorded if participants had consumed alcoholic beverages within the past 30 days [33].

### 2.5. Statistical Analysis

Numerical variables were summarized using two distinct approaches based on their distributional properties: means accompanied by standard deviations (SD) were used for normally distributed data, while medians with interquartile ranges (IQR) were reported for data that deviated from normality. Categorical variables were expressed as counts and percentages. For normally distributed data, comparisons between groups were performed using independent *t*-tests for two groups and analysis of variance (ANOVA) for multiple groups. Non-normally distributed data were analyzed using the Mann–Whitney U test for two groups and the Kruskal–Wallis test for multiple groups. Categorical comparisons were conducted using the chi-square test. Participants were categorized into four groups according to NHHR quartiles: the first quartile (Q1), second quartile (Q2), third quartile (Q3), and fourth quartile (Q4). To reduce potential confounding and achieve balanced group characteristics, propensity score matching (PSM) was performed, with standardized mean differences (SMD) used to assess balance between CKD and non-CKD participants (Table S1). To investigate the association between NHHR and CKD, multivariable logistic regression models were utilized. Restricted cubic splines (RCS) were applied to examine potential non-linear relationships between NHHR and CKD, with a piecewise logistic regression model to further explore this relationship. Three regression models were constructed: Model 1 was unadjusted and included no covariates; Model 2 adjusted for age and sex; and Model 3 incorporated additional adjustments for education, marital status, residence, body mass index, serum uric acid, triglyceride, hemoglobin A1c and fasting plasma glucose, systolic blood pressure, diastolic blood pressure, smoking, alcohol drinking, routine exercise, and diabetes duration. To assess potential multicollinearity in our dataset, the generalized variance inflation factor (GVIF) was calculated for all included variables. The results confirmed no significant multicollinearity (all GVIF^1/2Df^ < 2) (Table S2). Generalized additive models (GAMs) were employed to assess the relationships between NHHR and both UACR and eGFR. Furthermore, subgroup analyses were performed to evaluate the consistency of the NHHR–CKD association across various strata, including age, sex, BMI, hypertension status, glycemic control, smoking, and alcohol drinking status. The ROC curve was utilized to assess NHHR’s capability in differentiating CKD patients from non-CKD individuals, quantified by the area under curve (AUC). The DeLong test was applied to compare the diagnostic performance across various markers. Statistical significance was defined as a two-sided *p*-value of less than 0.05. To account for multiple comparisons and determine statistical significance, the Bonferroni correction was applied, setting the adjusted significance threshold at α = 0.007 for subgroup interaction tests (calculated as 0.05 divided by 7 subgroups) [34]. All analyses were conducted using R software (version 4.2.1; R Foundation for Statistical Computing, Vienna, Austria).

## 3. Results

### 3.1. Basic Characteristics of Participants

As summarized in Table 1, the study comprised 1756 participants, stratified by CKD status into 485 individuals with CKD and 1271 without CKD. The mean age of the overall population was 57.23 ± 10.15 years, with participants in the CKD group being significantly older than those in the non-CKD group (*p* < 0.001). The sex distribution was equal, with 50.11% females and 49.89% males, and no significant sex-based differences were observed between the CKD and non-CKD groups (*p* > 0.05). The overall mean BMI was 24.76 ± 3.43 kg/m^2^, with the CKD group demonstrating a significantly higher BMI compared to the non-CKD group (*p* < 0.001). Hypertension was markedly more prevalent in the CKD group than in the non-CKD group (*p* < 0.001). Further analysis of lipid profiles revealed significant disparities, including higher TG, TC, non-HDL-C levels and lower HDL-C levels in the CKD group compared to the non-CKD group (all *p* < 0.001). Glycemic control was notably worse in the CKD group, as evidenced by elevated HbA1c levels and FPG concentrations (both *p* < 0.001). Additionally, SUA levels were significantly higher in the CKD group (*p* < 0.001). Regarding lifestyle factors, alcohol consumption was less common in the CKD group compared to the non-CKD group, while no significant differences were observed in smoking or routine exercise between the two groups (all *p* > 0.05).

Table 2 presents the basic characteristics of participants stratified by NHHR quartiles. Significant differences were observed across quartiles for age, BMI, HDL-C, non-HDL-C, SUA levels, diabetes duration, and UACR (all *p* < 0.05). Additionally, the prevalence of elevated HbA1c, FPG, smoking, and CKD varied significantly (all *p* < 0.01), with the highest rates observed in the fourth quartile (Q4). In contrast, no significant differences were found for sex, educational attainment, residence, alcohol consumption, routine exercise, or eGFR distribution across the NHHR quartiles (all *p* > 0.05).

### 3.2. Multivariable Regression Analysis of NHHR Quartiles in Relation to CKD

To evaluate the relationship between NHHR and CKD, multivariable logistic regression models were employed. Odds ratios (ORs) and their 95% confidence intervals (CIs) were computed for NHHR quartiles, using Q1 as the reference group (Table 3). Initially, NHHR was analyzed as a continuous variable. In the unadjusted model (Model 1), each SD increase in NHHR was associated with ORs of 1.34 (1.21–1.49) in the overall population, 1.38 (1.19–1.60) in females, and 1.32 (1.14–1.53) in males. After adjusting for age and sex, the ORs remained largely unchanged. Further adjustments for covariates such as education, marital status, residence, body mass index, serum uric acid, triglyceride, hemoglobin A1c, fasting plasma glucose, systolic and diastolic blood pressure, smoking, alcohol consumption, physical activity, and diabetes duration yielded ORs of 1.23 (1.09–1.37) in the overall population, 1.22 (1.05–1.45) in females, and 1.25 (1.03–1.47) in males.

When NHHR was assessed categorically, the unadjusted model showed ORs for CKD of 1.36 (0.99–1.87) for Q2, 1.53 (1.12–2.09) for Q3, and 2.26 (1.67–3.05) for Q4 compared to Q1 in the overall population. In females, the ORs were 1.11 (0.71–1.73), 1.16 (0.75–1.81), and 2.16 (1.42–3.27), while in males, they were 1.57 (1.00–2.49), 2.06 (1.32–3.12), and 2.48 (1.59–3.85). After adjusting for age and sex in Model 2, the ORs exhibited minimal changes. In the fully adjusted Model 3, the ORs for Q2, Q3, and Q4 were 1.29 (0.92–1.79), 1.31 (0.94–1.83), and 1.87 (1.34–2.60), respectively, in the overall population. For females, the ORs were 0.95 (0.59–1.52), 1.08 (0.68–1.72), and 1.77 (1.13–2.79), while for males, they were 1.62 (1.00–2.64), 1.86 (1.15–3.03), and 2.19 (1.34–3.60). Significant trends were observed across all models in the overall population, females, and males (all *p* for trend < 0.05). The multivariable regression analysis conducted on the dataset after PSM further confirmed the robustness of these results (Table S3).

### 3.3. RCS Analysis of the Association Between NHHR and CKD

**Figure 2:**
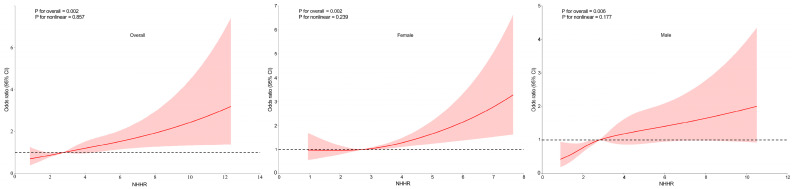
Association between the non-high-density lipoprotein cholesterol (non-HDL-C) to HDL-C ratio (NHHR) and chronic kidney disease (CKD) in overall population, females and males, with 95% confidence intervals (CIs). The figure depicts the association between NHHR and CKD, considering potential nonlinear associations. X-axis: Represents the levels of NHHR. Y-axis: Represents the odds ratios for CKD, indicating the relative likelihood of CKD presence associated with different levels of NHHR. The solid lines represent the fitted curves illustrating the association between NHHR and CKD. The dotted horizontal lines denote an OR of 1, representing the absence of association. The red areas surrounding the fitted curves indicate the 95% CIs for the predicted ORs. Adjusted for age, sex, education, marital status, residence, body mass index, serum uric acid, triglyceride, hemoglobin A1c and fasting plasma glucose, systolic blood pressure, diastolic blood pressure, smoking, alcohol drinking, routine exercise, and diabetes duration.

To explore potential non-linear relationships between NHHR and CKD, RCS analysis was conducted, with the results presented in Figure 2. After adjusting for covariates, a consistent linear dose–response relationship was observed between NHHR and CKD in the overall population, as well as in both females and males (all *p* for nonlinearity > 0.05). This suggests that higher NHHR levels are associated with an increased likelihood of CKD. Additionally, a linear relationship between NHHR and CKD was observed in the dataset after PSM (Figure S1). Furthermore, a piecewise regression analysis was conducted to evaluate the threshold effect of NHHR on CKD. The log-likelihood ratio test yielded a *p*-value > 0.05, suggesting that the relationship between NHHR and CKD does not significantly deviate from a linear model (Table S4).

### 3.4. GAM Analysis of NHHR with UACR and eGFR

**Figure 3:**
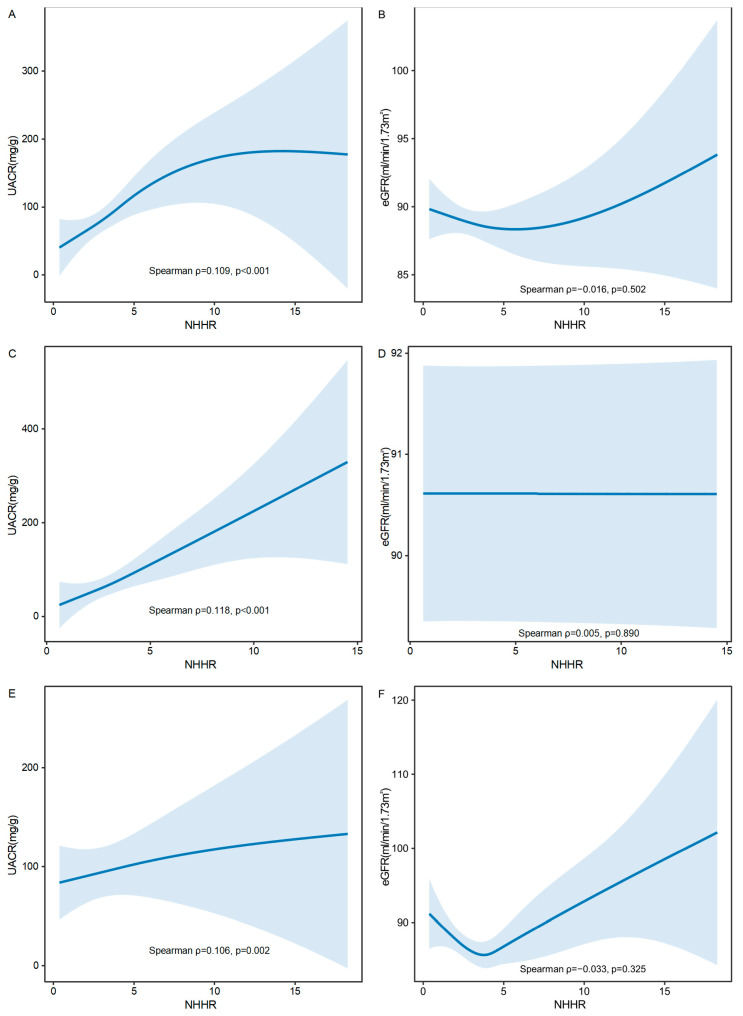
Correlation of the non-high-density lipoprotein cholesterol to high-density lipoprotein cholesterol ratio (NHHR) with both the urinary albumin-to-creatinine ratio (UACR) and the estimated glomerular filtration rate (eGFR), with 95% confidence intervals (CIs). (**A**) Correlation of NHHR with UACR in overall population. (**B**) Correlation of NHHR with eGFR in overall population. (**C**) Correlation of NHHR with UACR in females. (**D**) Correlation of NHHR with eGFR in females. (**E**) Correlation of NHHR with UACR in males. (**F**) Correlation of NHHR with eGFR in males. X-axis: Represents the levels of NHHR. Higher values on this axis indicate higher NHHR levels. Y-axis: Represents the levels of UACR or eGFR. The solid lines represent the fitted curves illustrating the trend of the relationship between NHHR with UACR and eGFR. Blue areas: Represent the 95% CIs around the fitted curves, indicating the precision of the estimated relationships. UACR, urinary albumin-to-creatinine ratio; eGFR, estimated glomerular filtration rate.

To further investigate these relationships, GAMs were used to evaluate the associations of NHHR with both UACR and eGFR in the overall population, as well as in females and males separately. As shown in Figure 3A,C,E, a statistically significant positive correlation was observed between NHHR and UACR (mg/g), with Spearman correlation coefficients of ρ = 0.109 (*p* < 0.001) in the overall population, ρ = 0.118 (*p* < 0.001) in females, and ρ = 0.106 (*p* = 0.002) in males. The smooth-fitted curves demonstrated a modest upward trend, suggesting that higher NHHR levels may be associated with increased UACR values. In contrast, no statistically significant correlation was found between NHHR and eGFR (mL/min/1.73 m^2^) in the overall population (ρ = −0.016, *p* = 0.502; Figure 3B), females (ρ = 0.005, *p* = 0.850; Figure 3D), or males (ρ = −0.033, *p* = 0.325; Figure 3F), indicating no significant relationship between NHHR and eGFR.

### 3.5. Results of Subgroup Analyses

**Figure 4:**
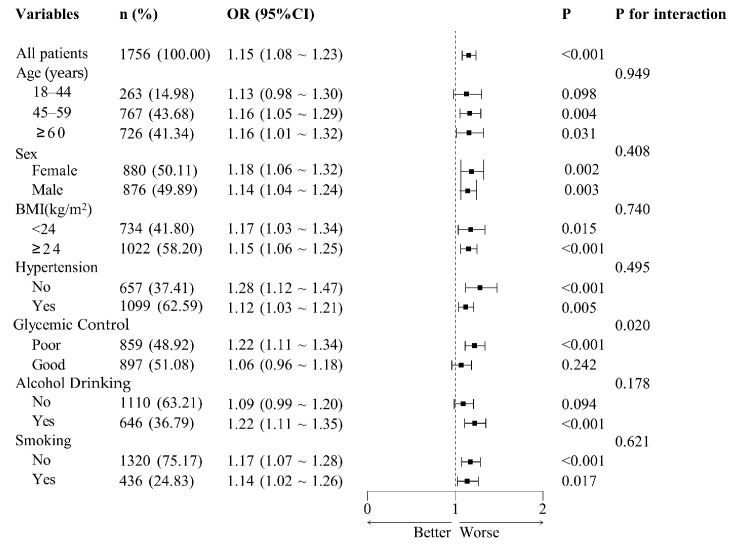
Subgroup analysis of adjusted odds ratios for non-high-density lipoprotein cholesterol to high-density lipoprotein cholesterol ratio (NHHR) and chronic kidney disease (CKD). Adjusted odds ratios (ORs) for the association between the NHHR and CKD across subgroups. Adjustments were made for age, sex, education, marital status, residence, body mass index, serum uric acid, triglyceride, hemoglobin A1c and fasting plasma glucose, systolic blood pressure, diastolic blood pressure, smoking, alcohol drinking, routine exercise, and diabetes duration. Each subgroup analysis excluded adjustments for the variable defining the subgroup. The central dashed line represents that OR = 1. Each horizontal bar represents the confidence interval (CI) for the OR of a subgroup. OR, odds ratio; BMI, body mass index.

Subgroup analyses were conducted across a range of demographic and clinical variables, including age categories (18–44 years, 45–59 years, and ≥60 years), sex (female or male), BMI (≥24 kg/m^2^ or <24 kg/m^2^), hypertension status (no or yes), glycemic control (poor or good), smoking status (no or yes), and alcohol consumption (no or yes) (Figure 4). The findings revealed statistically significant associations (all *p* < 0.05) in most subgroups, except for the 18–44 years age group, the good glycemic control group, and the non-alcohol drinking group, where the associations were not significant (*p* > 0.05). No significant interactions were detected between NHHR and the stratified variables, suggesting that the positive relationship between NHHR and CKD was consistent across all examined subgroups (all *p* for interaction > 0.007).

### 3.6. Roc Curve Assessment

**Figure 5:**
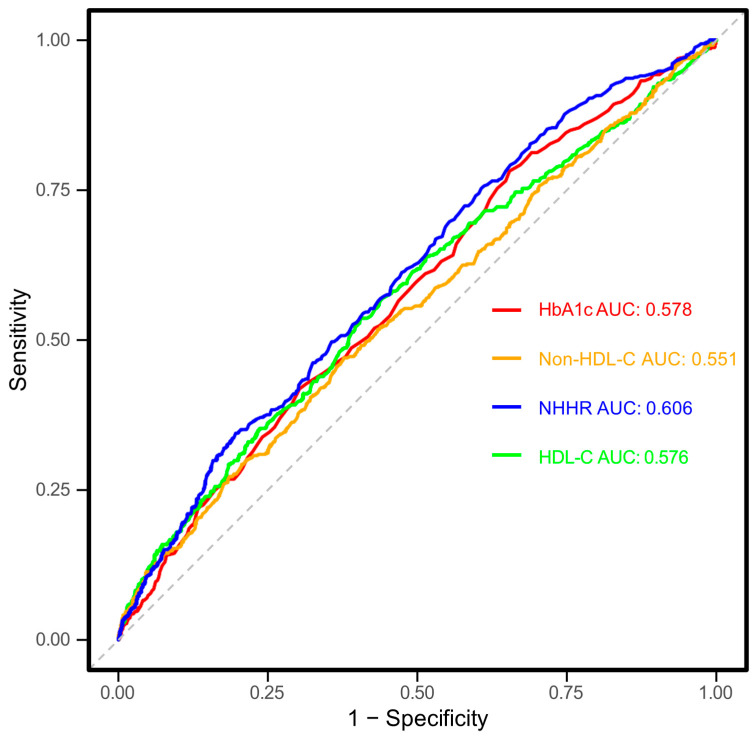
Receiver operating characteristic (ROC) curves for the association between non-high-density lipoprotein cholesterol (non-HDL-C) to high-density lipoprotein cholesterol ratio (NHHR), non-HDL-C, HDL-C and hemoglobin A1c (HbA1c) with chronic kidney disease (CKD). X-axis (1-Specificity): Represents the false-positive rate, indicating the proportion of individuals without CKD who are incorrectly classified as having CKD. Y-axis (Sensitivity): Represents the true-positive rate, indicating the proportion of individuals with CKD who are correctly classified as having CKD. The solid lines represent the ROC curves for each indicator, while the dashed line indicates the reference line representing no discriminatory ability. The area under the curve (AUC) values is provided for each indicator to quantify the discriminatory ability of NHHR for CKD. Higher AUC values (closer to 1) indicate better classification performance. HbA1c, hemoglobin A1c; HDL-C, high-density lipoprotein cholesterol; NHHR, non-HDL-C to HDL-C ratio; AUC, area under curve.

The ability of NHHR to diagnose CKD in T2DM individuals was evaluated using ROC curve analysis, as depicted in Figure 5. The analysis produced an AUC (95% CI) of 0.606 (0.577–0.635), indicating moderate diagnostic precision. The optimal threshold for NHHR was determined to be 3.48, with sensitivity and specificity values of 60.2% and 55.2%, respectively. Additionally, NHHR was compared with its individual elements (non-HDL-C and HDL-C) and HbA1c. The AUC (95% CI) values for non-HDL-C, HDL-C, and HbA1c were 0.551 (0.521–0.582), 0.576 (0.545–0.606), and 0.578 (0.548–0.607), respectively. According to the DeLong test, NHHR demonstrated significantly better predictive performance than HbA1c, non-HDL-C, and HDL-C (all *p* < 0.05), while no notable differences were observed among the other markers (all *p* > 0.05). These findings indicate that the composite NHHR offers greater diagnostic value.

## 4. Discussion

This study revealed a significant positive association between NHHR and CKD among Chinese individuals aged 18 years and older with T2DM. Higher NHHR levels and quartiles were linked to a progressively elevated prevalence of CKD, a finding reinforced by RCS and piecewise analyses, which demonstrated a linear dose–response relationship, both in females and males. After adjusting for covariates such as age, sex, education, marital status, residence, body mass index, serum uric acid, triglyceride, hemoglobin A1c and fasting plasma glucose, systolic blood pressure, diastolic blood pressure, smoking, alcohol drinking, routine exercise, and diabetes duration, the association remained significant (*p* < 0.05) across most subgroups. However, no significant associations were observed in the 18–44 years age group, the good glycemic control group, or the non-alcohol drinking group (*p* > 0.05). GAMs further confirmed a significant positive correlation between NHHR and UACR (ρ = 0.109, *p* < 0.001). ROC curve analysis demonstrated NHHR’s diagnostic potential, with an optimal cut-off value of 3.48 showing better predictive performance compared to its individual components and HbA1c. Sensitivity analyses conducted on the PSM-adjusted dataset further reinforced the robustness and reliability of our primary findings. These results highlight NHHR’s promise as a biomarker for assessing CKD risk in patients with T2DM.

The positive linear association between NHHR and CKD may be explained by several interrelated mechanisms, including atherogenic lipid metabolism abnormalities, oxidative stress, chronic inflammation, IR, sympathetic overactivity, lipotoxicity in renal tubules, and microvascular damage leading to fibrosis. These factors collectively contribute to renal impairment and CKD progression. Elevated NHHR reflects an imbalance between atherogenic lipoproteins (non-HDL-C) and protective lipoproteins (HDL-C), indicating a pro-atherogenic state. Elevated non-HDL-C, particularly LDL-C and VLDL-C, can penetrate the glomerular capillary wall, undergo oxidation (oxLDL), and trigger macrophage infiltration and foam cell formation, promoting glomerulosclerosis [35]. Simultaneously, reduced HDL-C impairs cholesterol efflux, weakens endothelial protection, and accelerates renal vascular dysfunction. This imbalance contributes to endothelial nitric oxide deficiency, leading to renal hypoperfusion and chronic hypoxia, further exacerbating CKD.

Oxidative stress and inflammation play a central role in NHHR-induced renal damage. oxLDL activates the NF-κB pathway, increasing pro-inflammatory cytokines such as TNF-α, IL-6, and IL-1β, which drive tubulointerstitial inflammation and fibrosis [36]. Meanwhile, HDL-C possesses anti-inflammatory and antioxidant properties, which are diminished in individuals with a high NHHR. This chronic inflammatory state disrupts tubuloglomerular feedback, leading to proteinuria and progressive nephron loss [37].

NHHR is also closely linked to IR, a key driver of CKD. IR contributes to glomerular hyperfiltration by impairing afferent arteriolar relaxation and increasing tubular sodium reabsorption, leading to intraglomerular hypertension [38]. Additionally, IR dysregulates lipid metabolism, promoting the overproduction of VLDL and small dense LDL-C while reducing HDL-C levels, further elevating NHHR [39]. These metabolic disturbances exacerbate oxidative stress and renal endothelial dysfunction, accelerating CKD progression. Sympathetic nervous system overactivity may further mediate the NHHR–CKD relationship. Increased sympathetic activity enhances renin–angiotensin–aldosterone system (RAAS) activation, raising glomerular pressure and worsening renal injury [40]. Lipotoxicity in renal tubular cells provides another potential link between NHHR and CKD. Elevated free fatty acids in a high-NHHR state accumulate in tubular epithelial cells, inducing mitochondrial dysfunction and apoptosis [41]. Lipid peroxidation products such as 4-hydroxynonenal contribute to tubular injury and interstitial fibrosis, exacerbating renal decline [42]. These effects may explain the observed association between NHHR and increased UACR, an early marker of renal damage. Microvascular injury and fibrosis may also underlie the NHHR–CKD association. Increased non-HDL-C promotes transforming growth factor-β1 expression, driving renal fibrosis [43]. Reduced HDL-C impairs cholesterol efflux from mesangial cells, facilitating mesangial expansion and glomerulosclerosis [44]. Persistent oxidative stress, inflammation, and IR further accelerate extracellular matrix deposition, leading to irreversible renal impairment [45].

The significant positive correlation between NHHR and UACR, but not eGFR, suggests that NHHR primarily reflects early glomerular and endothelial dysfunction rather than overt reductions in filtration capacity. UACR is a sensitive marker of glomerular endothelial injury and increased permeability, both of which are strongly influenced by lipid abnormalities [46]. A high NHHR indicates an imbalance favoring atherogenic lipoproteins (non-HDL-C) over protective HDL-C, leading to oxidative stress, inflammation, and endothelial dysfunction. oxLDL promotes podocyte injury and glomerular basement membrane thickening, increasing albumin leakage [47]. Additionally, impaired HDL function reduces cholesterol efflux from renal cells, exacerbating lipid accumulation and inflammatory cytokine release, further driving microvascular damage and albuminuria [48]. In contrast, eGFR reflects overall renal filtration capacity, which tends to decline later in CKD progression [49]. In early kidney dysfunction, compensatory hyperfiltration can mask reductions in eGFR despite ongoing glomerular damage. This compensatory response is particularly pronounced in diabetes, where intraglomerular hypertension initially preserves filtration despite endothelial injury [50]. Furthermore, eGFR estimation is influenced by muscle mass and hydration status, potentially reducing its sensitivity to NHHR-related microvascular injury [51].

The lack of a significant association between NHHR and CKD in the 18–44 years age group, the good glycemic control group, and the non-alcohol drinking group may be attributed to a combination of physiological resilience, metabolic stability, compensatory renal mechanisms, and the influence of additional protective factors in these subpopulations. Younger individuals (18–44 years) generally have greater physiological resilience and renal reserve, which may mitigate the adverse effects of dyslipidemia on kidney function [52]. Renal aging involves progressive structural and functional changes, including reduced nephron number, glomerular hypertrophy, and increased susceptibility to oxidative stress and inflammation [53]. In contrast, younger individuals have a more robust capacity for glomerular autoregulation and lipid metabolism [54], which may buffer the detrimental impact of an elevated NHHR. In individuals with good glycemic control, this may be due to lower systemic metabolic stress and reduced vascular damage. Hyperglycemia exacerbates dyslipidemia-induced renal injury by promoting advanced glycation end-products, increasing oxidative stress, and activating the RAAS [55]. In contrast, patients with well-controlled blood glucose levels experience less endothelial dysfunction, lower inflammation, and reduced lipid oxidation, which may attenuate the nephrotoxic effects of NHHR [56]. Moreover, proper glycemic control may help maintain HDL-C functionality, preserving its anti-inflammatory, antioxidant, and endothelial-protective properties, thereby weakening the NHHR–CKD association in this subgroup [57]. The lack of an association in the non-alcohol drinking group suggests a potential modifying effect of alcohol consumption on lipid metabolism and renal function. Non-alcohol drinkers may exhibit healthier lifestyle patterns that mitigate the adverse effects of dyslipidemia on renal health. In contrast, heavy alcohol consumption may contribute to lipid abnormalities, hypertension, and oxidative stress, which may exacerbate CKD risk in those with an elevated NHHR [58].

Our results align with prior research highlighting the significant association between NHHR and renal function. For instance, Pan et al. analyzed data from the National Health and Nutrition Examination Survey (NHANES) between 2005 and 2016, involving 4177 diabetic patients, and identified a positive but non-linear relationship between NHHR and diabetic kidney disease (DKD) [59]. Similarly, Zhang et al., using NHANES data from 1999 to 2020 with 8329 diabetic participants, also reported a non-linear positive correlation between NHHR and DKD [60]. Further corroborating these findings, Huang et al. examined data from 19,458 individuals in the 1999–2018 NHANES and found that higher NHHR levels were associated with an increased likelihood of macroalbuminuria in U.S. adults [61]. Additionally, Hong et al. demonstrated that elevated NHHR levels were significantly linked to a higher risk of kidney stone development and recurrence in the U.S. adult population [62]. Du et al. further supported this by suggesting a strong connection between increased NHHR levels and a heightened risk of kidney stones among American adults [63].

This study has several notable strengths. First, it represents the first investigation into the association between NHHR and CKD in a well-characterized sample of Chinese adults with T2DM, providing critical insights into this relationship within a high-risk population. Second, the use of standardized data collection methods enhances the reliability and consistency of the findings.

However, certain limitations should be acknowledged. The study population was limited to T2DM patients from Eastern China, which may restrict the generalizability of the results to other regions or demographic groups. Although extensive adjustments were made for various confounders, the possibility of residual confounding cannot be entirely eliminated. Additionally, the analysis did not account for medication use, which could influence the observed outcomes. What is more, the reliance on eGFR calculated from serum creatinine, rather than direct measurement of creatinine clearance, may introduce systematic inaccuracies due to assumptions in the estimation formula or variability in creatinine production across individuals. This limitation is contextualized by the study’s alignment with a national diabetes complications investigation, which standardized eGFR calculation over direct creatinine measurement for consistency across sites. Finally, the cross-sectional nature of the study design precludes the establishment of causal relationships between the variables examined.

## 5. Conclusions

This study identifies a significant positive association between NHHR and CKD prevalence among Chinese adults with T2DM, suggesting that NHHR may serve as a complementary marker to existing clinical tools for identifying individuals at risk of early renal function deterioration in this population. However, longitudinal research is needed to confirm its predictive value for CKD onset and progression.

## Figures and Tables

**Table 1:** Participants’ basic characteristics by CKD status (*n* = 1756).

Characteristics	Total (*n* = 1756)	CKD Group (*n* = 485)	Non-CKD Group (*n* = 1271)	χ^2^/t/z	*p*
Sex, *n* (%)				0.048 ^b^	0.827
Female	880 (50.11)	241 (49.69)	639 (50.28)		
Male	876 (49.89)	244 (50.31)	632 (49.72)		
BMI, mean ± SD, kg/m^2^	24.76 ± 3.43	25.43 ± 3.56	24.51 ± 3.35	−5.06 ^a^	<0.001 ***
Age, mean ± SD, years	57.23 ± 10.15	59.09 ± 10.54	56.52 ± 9.92	−4.77 ^a^	<0.001 ***
Marriage status				0.58 ^b^	0.446
Married	1604 (91.34)	439 (90.52)	1165 (91.66)		
Others	152 (8.66)	46 (9.48)	106 (8.34)		
Educational attainment, *n* (%)				8.12 ^b^	0.017 *
Secondary education or below	1541 (87.76)	411 (84.74)	1130 (88.90)		
Senior high school	171 (9.74)	63 (12.99)	108 (8.50)		
College education or above	44 (2.50)	11 (2.27)	33 (2.60)		
Residence, *n* (%)				1.30 ^b^	0.255
Urban	881 (50.17)	254 (52.37)	627 (49.33)		
Rural	875 (49.83)	231 (47.63)	644 (50.67)		
Hypertension, *n* (%)	1099 (62.59)	385 (79.38)	714 (56.18)	80.73 ^b^	<0.001 ***
TG, median (IQR), mmol/L	1.60 (1.12–2.42)	1.87 (1.30–2.94)	1.51 (1.06–2.26)	47.47 ^c^	<0.001 ***
TC, mean ± SD, mmol/L	4.65 ± 1.07	4.78 ± 1.29	4.61 ± 0.97	−2.64 ^a^	0.009 **
HDL-C, mean ± SD, mmol/L	1.25 ± 0.36	1.18 ± 0.37	1.28 ± 0.35	4.96 ^a^	<0.001 ***
Non-HDL-C, mean ± SD, mmol/L	3.41 ± 1.09	3.60 ± 1.30	3.34 ± 0.99	−4.07 ^a^	<0.001 ***
LDL-C, mean ± SD, mmol/L	2.73 ± 0.90	2.70 ± 1.02	2.75 ± 0.85	0.98 ^a^	0.325
HbA1c, mean ± SD, %	7.27 ± 1.49	7.61 ± 1.65	7.14 ± 1.40	−5.43 ^a^	<0.001 ***
FPG, mean ± SD, mmol/L	7.94 ± 2.58	8.54 ± 3.11	7.72 ± 2.31	−5.32 ^a^	<0.001 ***
SUA, mean ± SD, μmol/L	5.63 ± 1.59	6.01 ± 1.87	5.48 ± 1.44	−5.66 ^a^	<0.001 ***
eGFR, mean ± SD, mL/min/1.73 m^2^	88.92 ± 19.03	78.76 ± 25.61	92.80 ± 14.01	11.44 ^a^	<0.001 ***
UACR, median (IQR), mg/g	10.88 (4.83, 28.26)	62.62 (37.25, 159.50)	7.18 (3.85, 13.24)	−28.60 ^c^	<0.001 ***
Diabetes duration, median(IQR), years	6.00 (3.00, 10.00)	7.00 (4.00, 13.00)	6.00 (3.00, 10.00)	−4.73 ^c^	<0.001 ***
Smoking, *n* (%)	436 (24.83)	118 (24.33)	318 (25.02)	0.09 ^b^	0.765
Alcohol drinking, *n* (%)	646 (36.79)	160 (32.99)	486 (38.24)	4.16 ^b^	0.042 *
Routine exercise, *n* (%)	290 (16.51)	74 (15.26)	216 (16.99)	0.77 ^b^	0.381
NHHR, median (IQR)	2.76 (2.02, 3.66)	2.99 (2.23, 4.04)	2.66 (1.94, 3.49)	−5.69 ^c^	<0.001 ***

^a^ Student’s *t*-test; ^b^ Chi-square test; ^c^ Mann–Whitney U test. * *p* < 0.05; ** *p* < 0.01; *** *p* < 0.001. Abbreviations: CKD, chronic kidney disease; SD, standard deviation; BMI, body mass index; IQR, interquartile range; TG, triglycerides; LDL-C, low-density lipoprotein cholesterol; TC, total cholesterol; HDL-C, high-density lipoprotein cholesterol; Non-HDL-C, non-high-density lipoprotein cholesterol; HbA1c, hemoglobin A1c; FPG, fasting plasma glucose; SUA, serum uric acid; UACR, urinary albumin-to-creatinine ratio; eGFR, estimated glomerular filtration rate; NHHR, non-high-density lipoprotein cholesterol to high-density lipoprotein cholesterol ratio.

**Table 2:** Participants’ basic characteristics by NHHR quartile (*n* = 1756).

Variables	Total (*n* = 1756)	Q1 (*n* = 439)	Q2 (*n* = 439)	Q3 (*n* = 439)	Q4 (*n* = 439)	χ^2^/F	*p*
Sex, *n* (%)						6.23 ^a^	0.101
Female	880 (50.11)	219 (49.89)	231 (52.62)	231 (52.62)	199 (45.33)		
Male	876 (49.89)	220 (50.11)	208 (47.38)	208 (47.38)	240 (54.67)		
Age, Mean ± SD, years	57.23 ± 10.15	57.87 ± 9.82	58.31 ± 9.78	57.14 ± 10.13	55.59 ± 10.68	6.12 ^b^	<0.001 ***
Educational attainment, *n* (%)						11.59 ^a^	0.072
Secondary education or lower	1541 (87.76)	396 (90.21)	396 (90.21)	379 (86.33)	370 (84.28)		
Senior high school	171 (9.74)	32 (7.29)	36 (8.20)	48 (10.93)	55 (12.53)		
College education or above	44 (2.51)	11 (2.51)	7 (1.59)	12 (2.73)	14 (3.19)		
Residence, *n* (%)						7.64 ^a^	0.054
Urban	875 (49.83)	234 (53.30)	228 (51.94)	217 (49.43)	196 (44.65)		
Rural	881 (50.17)	205 (46.70)	211 (48.06)	222 (50.57)	243 (55.35)		
BMI, Mean ± SD, kg/m^2^	24.76 ± 3.43	23.41 ± 3.35	24.48 ± 3.24	25.31 ± 3.17	25.84 ± 3.48	45.00 ^b^	<0.001 ***
HDL-C, mean ± SD, mmol/L	1.25 ± 0.36	1.60 ± 0.36	1.32 ± 0.25	1.15 ± 0.20	0.93 ± 0.20	528.98 ^b^	<0.001 ***
Non-HDL-C, mean ± SD, mmol/L	3.41 ± 1.09	2.41 ± 0.58	3.14 ± 0.61	3.64 ± 0.63	4.44 ± 1.20	501.15 ^b^	<0.001 ***
SUA, mean ± SD, μmol/L	334.65 ± 94.44	309.93 ± 89.57	329.18 ± 91.91	336.56 ± 87.71	362.92 ± 100.63	24.65 ^b^	<0.001 ***
Elevated HbA1c, *n* (%)	859 (48.92)	181 (41.23)	190 (43.28)	224 (51.03)	264 (60.14)	38.86 ^a^	<0.001 ***
Elevated FPG, *n* (%)	989 (56.32)	229 (52.16)	226 (51.48)	259 (59.00)	275 (62.64)	15.67 ^a^	0.001 **
Hypertension, *n* (%)	1099 (62.59)	237 (53.99)	271 (61.73)	285 (64.92)	306 (69.70)	24.52 ^a^	<0.001 ***
Smoking, *n* (%)	436 (24.83)	96 (21.87)	93 (21.18)	102 (23.23)	145 (33.03)	21.60 ^a^	<0.001 ***
Alcohol drinking, *n* (%)	646 (36.79)	174 (39.64)	155 (35.31)	160 (36.45)	157 (35.76)	2.16 ^a^	0.539
Routine exercise, *n* (%)	290 (16.51)	65 (14.81)	74 (16.86)	80 (18.22)	71 (16.17)	1.93 ^a^	0.586
Diabetes duration, median (IQR), years	6.00 (3.00, 10.00)	7.00 (4.00, 11.00)	6.00 (3.00, 10.00)	6.00 (3.00, 10.00)	5.00 (2.00, 10.00)	11.10 ^c^	0.011 *
UACR, median (IQR), mg/g	10.88 (4.83, 28.26)	9.80 (4.26, 20.81)	10.47 (4.76, 25.26)	10.66 (4.95, 31.64)	12.69 (5.40, 43.10)	18.09 ^c^	<0.001 ***
eGFR, mean ± SD, mL/min/1.73 m^2^	88.92 ± 19.03	90.18 ± 16.97	88.20 ± 18.87	89.65 ± 18.50	87.66 ± 21.47	1.71 ^b^	0.162
CKD, *n* (%)	485 (27.62)	89 (20.27)	113 (25.74)	123 (28.02)	160 (36.45)	29.77 ^a^	<0.001 ***

^a^ Chi-square test; ^b^ ANOVA; ^c^ Kruskal–Waills test. * *p* < 0.05; ** *p* < 0.01; *** *p* < 0.001. Abbreviations: NHHR, non-high-density lipoprotein cholesterol to high-density lipoprotein cholesterol ratio; Q1, first quartile; Q2, second quartile; Q3, third quartile; Q4, fourth quartile; BMI, body mass index; HDL-C, high-density lipoprotein cholesterol; Non-HDL-C, non-high-density lipoprotein cholesterol; SUA, serum uric acid; HbA1c, hemoglobin A1c; FPG, fasting plasma glucose; UACR, urinary albumin-to-creatinine ratio; eGFR, estimated glomerular filtration rate; CKD, chronic kidney disease.

**Table 3:** Multivariable regression analysis of NHHR quartiles in relation to CKD (*n* = 1756).

	Model 1		Model 2		Model 3	
	OR (95% CI)	*p*	OR (95% CI)	*p*	OR (95% CI)	*p*
NHHR per SD	1.34 (1.21–1.49)	<0.001 ***	1.40 (1.26–1.56)	<0.001 ***	1.23 (1.09–1.37)	<0.001 ***
NHHR quartile						
Q1 (*n* = 439)	1.00 (ref)		1.00 (ref)		1.00 (ref)	
Q2 (*n* = 439)	1.36 (0.99–1.87)	0.055	1.37 (0.99–1.88)	0.056	1.29 (0.92–1.79)	0.135
Q3 (*n* = 439)	1.53 (1.12–2.09)	0.008 **	1.59 (1.16–2.18)	0.004 **	1.31 (0.94–1.83)	0.110
Q4 (*n* = 439)	2.26 (1.67–3.05)	<0.001 ***	2.45 (1.80–3.33)	<0.001 ***	1.87 (1.34–2.60)	<0.001 ***
*p* for trend	1.32 (1.19–1.46)	<0.001 ***	1.36 (1.23–1.51)	<0.001 ***	1.21 (1.08–1.36)	0.001 **
Female						
NHHR per SD	1.38 (1.19–1.60)	<0.001 ***	1.43 (1.23–1.66)	<0.001 ***	1.22 (1.05–1.45)	0.011 *
NHHR quartile						
Q1 (*n* = 220)	1.00 (ref)		1.00 (ref)		1.00 (ref)	
Q2 (*n* = 220)	1.11 (0.71–1.73)	0.651	1.17 (0.75–1.84)	0.495	0.95 (0.59–1.52)	0.819
Q3 (*n* = 220)	1.16 (0.75–1.81)	0.501	1.19 (0.76–1.86)	0.452	1.08 (0.68–1.72)	0.736
Q4 (*n* = 220)	2.16 (1.42–3.27)	<0.001 ***	2.32 (1.51–3.55)	<0.001 ***	1.77 (1.13–2.79)	0.013 *
*p* for trend	1.34 (1.15–1.56)	<0.001 ***	1.37 (1.18–1.61)	<0.001 ***	1.24 (1.05–1.47)	0.010 *
Male						
NHHR per SD	1.32 (1.14–1.53)	<0.001 ***	1.39 (1.19–1.61)	<0.001 ***	1.25 (1.03–1.47)	0.008 **
NHHR quartile						
Q1 (*n* = 219)	1.00 (ref)		1.00 (ref)		1.00 (ref)	
Q2 (*n* = 219)	1.57 (1.00–2.49)	0.051	1.58 (1.00–2.50)	0.050	1.62 (1.00–2.64)	0.050
Q3 (*n* = 219)	2.06 (1.32–3.12)	0.002 **	2.14 (1.37–3.36)	<0.001 ***	1.86 (1.15–3.03)	0.012 *
Q4 (*n* = 219)	2.48 (1.59–3.85)	<0.001 ***	2.75 (1.76–4.31)	<0.001 ***	2.19 (1.34–3.60)	0.002 **
*p* for trend	1.33 (1.16–1.52)	<0.001 ***	1.38 (1.20–1.58)	<0.001 ***	1.27 (1.08–1.48)	0.003 **

* *p* < 0.05; ** *p* < 0.01; *** *p* < 0.001. Abbreviations: NHHR, non-high-density lipoprotein cholesterol to high-density lipoprotein cholesterol ration; CKD, chronic kidney disease; OR, odds ratio; CI, confidence interval; Q1, first quartile; Q2, second quartile; Q3, third quartile; Q4, fourth quartile; ref, reference. Model 1: unadjusted analysis (no covariates included); Model 2: adjusted for age, sex; Model 3: fully adjusted model, incorporating age, sex, education, marital status, residence, body mass index, serum uric acid, triglyceride, hemoglobin A1c and fasting plasma glucose, systolic blood pressure, diastolic blood pressure, smoking, alcohol drinking, routine exercise, and diabetes duration.

## Data Availability

The data presented in this study are available on request from the corresponding author. The data are not publicly available due to privacy restrictions.

## Supplementary Materials

The following supporting information can be downloaded at: https://www.mdpi.com/article/10.3390/nu17071125/s1, Table S1: Covariates before and after PSM. Table S2: Assessment of multicollinearity among independent variables (*n* = 1756). Table S3: Multivariable regression analysis of NHHR quartiles in relation to CKD after PSM (*n* = 970). Table S4: Threshold effect analysis of NHHR on CKD (*n* = 970). Figure S1: Association between the non-high-density lipoprotein cholesterol (non-HDL-C) to HDL-C ratio (NHHR) and chronic kidney disease (CKD), with 95% confidence intervals (CIs).

## Abbreviations

The following abbreviations are used in this manuscript:

HDL-C	High-density lipoprotein cholesterol
Non-HDL-C	Non-high-density lipoprotein cholesterol
NHHR	Non-high-density lipoprotein cholesterol to high-density lipoprotein cholesterol ratio
T2DM	Type 2 diabetes mellitus
CKD	Chronic kidney disease
RCS	Restricted cubic spline
GAM	Generalized additive models
UACR	Urinary albumin-to-creatinine ratio
eGFR	Estimated glomerular filtration rate
BMI	Body mass index
TC	Total cholesterol
TG	Triglycerides
SUA	Serum uric acid
LDL-C	Low-density lipoprotein cholesterol
VLDL-C	Very-low-density lipoprotein cholesterol
IR	Insulin resistance
HbA1c	Hemoglobin A1c
FPG	Fasting blood glucose
SD	Standard deviation
IQR	Interquartile range
OR	Odds ratio
CI	Confidence interval
Q1	First quartile
Q2	Second quartile
Q3	Third quartile
Q4	Fourth quartile
PSM	Propensity score matching
SMD	Standardized mean difference
ANOVA	Analysis of variance
RAAS	Renin-angiotensin-aldosterone system
NHANES	National Health and Nutrition Examination Survey
