# Supplementary material for: The Non-High-Density Lipoprotein Cholesterol (Non-HDL-C) to HDL-C Ratio (NHHR) and Its Association with Chronic Kidney Disease in Chinese Adults with Type 2 Diabetes: A Preliminary Study
Source: Nutrients. 2025 Mar 24;17(7):1125. doi: 10.3390/nu17071125 (PMC11990853; doi:10.3390/nu17071125)
Supplement: Supplementary file 1 [file nutrients-17-01125-s001.zip › nutrients-3535027-supplementary.pdf]

Supplementary: **Table S1. Covariates before and after PSM**

Variables	Level	Before Matching			After Matching		
		Non-CKD group	CKD group	SMD	Non-CKD group	CKD group	SMD
n		1269	485		485	485	
BMI (mean (SD)), kg/m^2		24.51 (3.35)	25.43 (3.56)	0.257	25.20 (3.52)	25.43 (3.56)	0.065
HbA1c (mean (SD))		7.14 (1.40)	7.61 (1.65)	0.285	7.47 (1.50)	7.61 (1.65)	0.086
TG (mean (SD)), mmol/L		2.01 (2.12)	2.80 (3.61)	0.219	2.46 (3.05)	2.80 (3.61)	0.094
SUA (mean (SD)), mmol/L		325.96 (85.47)	357.60 (111.48)	0.284	352.27 (90.10)	357.60 (111.48)	0.048
Age (mean (SD)), years		56.52 (9.92)	59.09 (10.54)	0.243	59.00 (9.16)	59.09 (10.54)	0.008
Sex (%)	Female	637 (50.2)	241 (49.7)	-0.010	243 (50.1)	241 (49.7)	-0.008
	Male	632 (49.8)	244 (50.3)	0.010	242 (49.9)	244 (50.3)	0.008
Smoking (%)	No	951 (74.9)	367 (75.7)	0.017	371 (76.5)	367 (75.7)	-0.019
	Yes	318 (25.1)	118 (24.3)	-0.017	114 (23.5)	118 (24.3)	0.019
Alcohol drinking (%)	No	784 (61.8)	325 (67.0)	0.111	320 (66.0)	325 (67.0)	0.022
	Yes	485 (38.2)	160 (33.0)	-0.111	165 (34.0)	160 (33.0)	-0.022
FPG (mean (SD)), mmol/L		7.72 (2.31)	8.54 (3.11)	0.266	8.29 (2.65)	8.54 (3.11)	0.081
Education attainment (%)	Secondary education or below	1128 (88.9)	411 (84.7)	-0.115	427 (88.0)	411 (84.7)	-0.092
	Senior high school	108 (8.5)	63 (13.0)	0.133	53 (10.9)	63 (13.0)	0.061
	College education or above	33 (2.6)	11 (2.3)	-0.022	5 (1.0)	11 (2.3)	0.083
Marriage status (%)	Married	1163 (91.6)	439 (90.5)	-0.039	442 (91.1)	439 (90.5)	-0.021
	Others	106 (8.4)	46 (9.5)	0.039	43 (8.9)	46 (9.5)	0.021
Routine exercise (%)	No	1053 (83.0)	411 (84.7)	0.049	408 (84.1)	411 (84.7)	0.017
	Yes	216 (17.0)	74 (15.3)	-0.049	77 (15.9)	74 (15.3)	-0.017
SBP(mean (SD)), mm Hg		131.29 (16.27)	141.69 (18.51)	0.562	140.54 (15.75)	141.69 (18.51)	0.062
DBP (mean (SD)), mm Hg		76.60 (9.62)	79.58 (10.91)	0.273	79.11 (9.68)	79.58 (10.91)	0.043
Diabetes duration (mean (SD)), years		7.10 (5.68)	8.62 (6.23)	0.244	8.40 (6.25)	8.62 (6.23)	0.036
Residence (%)	Rural	643 (50.7)	231 (47.6)	-0.061	231 (47.6)	231 (47.6)	0.000
	Urban	626 (49.3)	254 (52.4)	0.061	254 (52.4)	254 (52.4)	0.000

Abbreviations: CKD, chronic kidney disease; PSM, propensity score matching; SMD,

standardized mean difference; BMI, body mass index; TG, triglycerides; HbA1c, hemoglobin A1c; FPG, fasting plasma glucose; SBP systolic blood pressure; DBP, diastolic blood pressure.

Table S2. Assessment of multicollinearity among independent variables (*n*=1,756)

Variables	GVIF	Df	$GVIF^{1/2Df}$
Age	1.48131	1	1.217091
BMI	1.230021	1	1.109063
NHHR	2.83724	1	1.684411
Sex	1.750792	1	1.323175
Marriage status	1.03333	1	1.016528
Residence	1.120586	1	1.058577
Education attainment	1.148141	2	1.035139
Smoking	1.519136	1	1.232532
Alcohol drinking	1.32128	1	1.149469
Routine exercise	1.01666	1	1.008296
Diabetes duration	1.136292	1	1.06597
TG	2.832503	1	1.683004
SBP	1.880363	1	1.371263
DBP	2.049352	1	1.431556
HbA1c	2.346103	1	1.531699
FPG	2.352911	1	1.53392
SUA	1.243297	1	1.115032

Abbreviations: NHHR, non-high-density lipoprotein cholesterol to high-density lipoprotein cholesterol ratio; BMI, body mass index; TG, triglycerides; HbA1c, hemoglobin A1c; FPG, fasting plasma glucose; SBP systolic blood pressure; DBP, diastolic blood pressure.

Table S3. Multivariable regression analysis of NHHR quartiles in relation to CKD after PSM (*n*=970)

	Model1		Model2		Model3	
	OR(95%CI)	*p*	OR(95%CI)	*p*	OR(95%CI)	*p*
NHHR (continuous)	1.09(1.01-1.17)	0.018	1.09(1.02-1.17)	<0.001	1.08(1.08-1.16)	0.044
NHHR quartile						
Q1 (n=243)	1.00(ref)		1.00(ref)		1.00(ref)	
Q2 (n=242)	1.13(0.79-1.62)	0.497	1.13(0.79-1.62)	0.501	1.19(0.82-1.74)	0.354
Q3 (n=242)	1.17(1.82-1.67)	0.389	1.18(1.82-1.67)	0.369	1.21(1.82-1.78)	0.334
Q4 (n=243)	1.73(1.21-2.48)	0.003	1.74(1.21-2.50)	0.003	1.76(1.19-2.61)	0.004
P for trend	1.20(1.07-1.35)	0.003	1.21(1.07-1.36)	0.002	1.21(1.07-1.38)	0.003

Abbreviations: NHHR, non-high-density lipoprotein cholesterol to high-density lipoprotein cholesterol ratio; CKD, chronic kidney disease; PSM, propensity score matching; OR, odds ratio; CI, confidence interval; Q1, first quartile; Q2, second quartile; Q3, third quartile; Q4, fourth quartile; ref, reference.

Model 1: unadjusted analysis (no covariates included); Model 2: adjusted for age, sex;

Model 3: fully adjusted model, incorporating age, sex, education, residence, marriage status, body mass index, triglyceride, serum uric acid; hemoglobin A1c, fasting plasma glucose, routine exercise; systolic blood pressure, diastolic blood pressure; smoking, alcohol drinking, and diabetes duration.

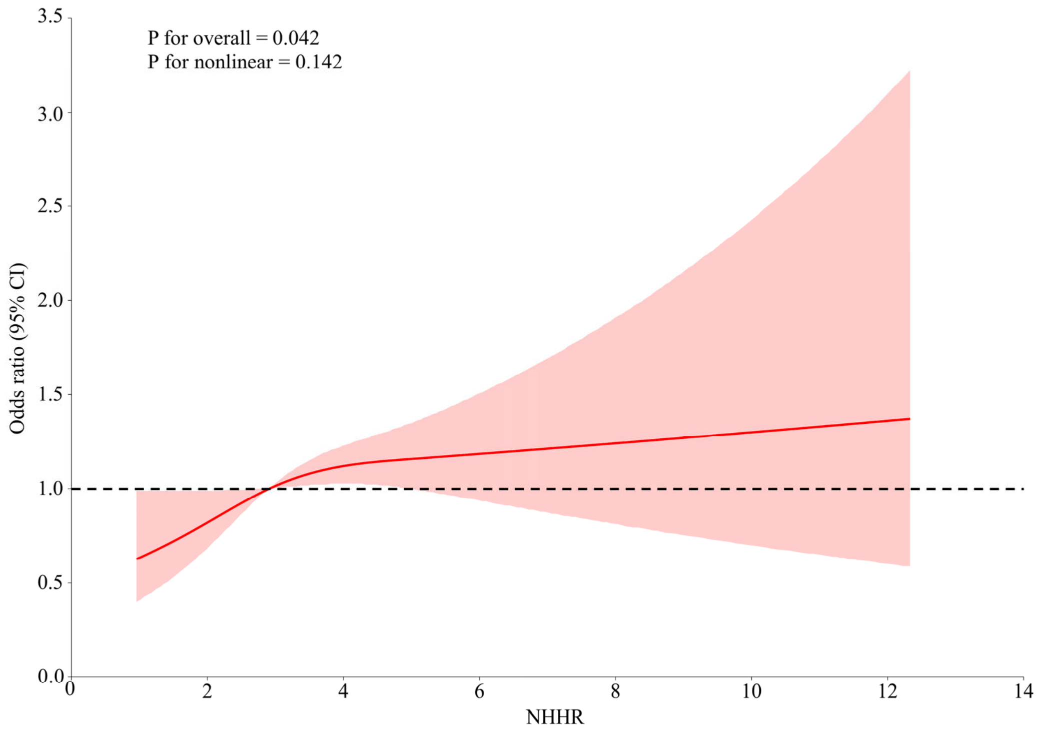

Figure S1. Association between the non-high-density lipoprotein cholesterol (non-HDL-C) to HDL-C ratio (NHHR) and chronic kidney disease (CKD), with 95% confidence intervals (CIs). The figure depicts the association between NHHR and CKD, considering potential nonlinear associations. X-axis: Represents the levels of NHHR. Y-axis: Represents the odds ratios for CKD, indicating the relative likelihood of CKD presence associated with different levels of NHHR. The solid lines represent the fitted curves illustrating the association between NHHR and CKD. The dotted horizontal lines denote an OR of 1, representing the absence of association. The shaded areas surrounding the fitted curves indicate the 95% CIs for the predicted ORs. Adjusted for age, sex, education, marital status, residence, body mass index, serum uric acid, triglyceride, hemoglobin A1c and fasting plasma glucose, systolic blood pressure, diastolic blood pressure, smoking, alcohol drinking, routine exercise and diabetes duration.

Table S4. Threshold efect analysis of NHHR on CKD (*n*=970)

	OR (95% CI)*	p
**Fitting by standard Logistic regression model**	1.17 (1.09 - 1.25)	<0.001
**Fitting by piecewise Logistic regression model (break-point = 5.47)**		
NHHR < 5.47	1.20 (1.08 - 1.35)	<0.001
NHHR≥ 5.47	1.05 (0.89 - 1.23)	0.586
**Log likelihood ratio**		**0.563**

Abbreviations: NHHR, non-high-density lipoprotein cholesterol to high-density lipoprotein cholesterol ratio; CKD, chronic kidney disease; OR, odds ratio; CI, confidence interval.

*Adjusted for age, sex, education, marital status, residence, body mass index, serum uric acid, triglyceride, hemoglobin A1c and fasting plasma glucose, systolic blood pressure, diastolic blood pressure, smoking, alcohol drinking, routine exercise and diabetes duration.
